# Model Predicts That MKP1 and TAB1 Regulate p38α Nuclear Pulse and Its Basal Activity through Positive and Negative Feedback Loops in Response to IL-1

**DOI:** 10.1371/journal.pone.0157572

**Published:** 2016-06-17

**Authors:** Raghvendra Singh

**Affiliations:** Department of Chemical Engineering, Indian Institute of Technology Kanpur, Kanpur, India; The University of Tokyo, JAPAN

## Abstract

Interleukin-1 mediates inflammation and stress response through nuclear activity of p38α. Although IL-1 receptor is not degraded, p38α activation is transient. IL-1 also causes cell migration and EMT by modulating cell-cell junctions. Although molecules involved in p38 activation are known, mechanism of the transient nuclear response and its basal activity remains unknown. By mathematical modeling of IL1/p38 signaling network, we show that IL-1 induces robust p38α activation both in the nucleus and in the cytoplasm/membrane. While nuclear response consists of an acute phase, membrane response resembles a step change. Following stimulation, p38α activity returns to a basal level in absence of receptor degradation. While nuclear pulse is controlled by MKP1 through a negative feedback to pp38, its basal activity is controlled by both TAB1 and MKP1 through a positive feedback loop. Our model provides insight into the mechanism of p38α activation, reason for its transient nuclear response, and explanation of the basal activity of MKK3/6 and p38α, which has been experimentally observed by other groups.

## Introduction

Proinflammatory cytokine interleukin-1 has been shown to activate multiple pathways such as JNK [1], NF-kB [2], and p38 [3] leading to transcription of proteins mediating inflammatory and stress response. The signaling starts with binding of IL-1 to its receptor IL1-RI and its accessory protein IL-1RAcP, causing intracellular complex formation involving myeloid differentiation factor, MyD88, and phosphorylation of IL-1 receptor associated kinase, IRAK [4, 5]. Phosphorylated IRAK dissociates from the receptor and binds TRAF6 [6–8]. IRAK-TRAF6 complex binds with TAB2 at the membrane, where IRAK is ubiquitinated and degraded [6, 8]. IRAK degradation leads to translocation of TAB2-TRAF6 complex to cytoplasm, which results in its binding to TAK1, causing TAK1 activation [6, 7]. TAK1 activation causes phosphorylation of MAP kinase kinase (MKK3/6), which activates p38 [9, 10]. Tyrosine-threonine phosphorylated p38 has been shown to mediate diverse cellular responses such as stress [11, 12], inflammation [11–13], migration [14, 15], differentiation [16, 17], and apoptosis [18, 19]. For responses requiring gene expression, p38 translocates to nucleus [20, 21] and activates transcription factors such as MEF2C, GADD153, SP1, AFT2 [12, 22–24]. On the other hand, for cell migration [25–27] and epithelial-to-mesenchymal transformation [28–30], which require modulation of adherens, tight and gap junctions, active p38 migrates to membrane [31–33] and regulates E-cadherin, claudin-1 and Cx32 [34–36]. Thus, both nuclear and membrane translocation of p38 may be required however the exact mechanism remains unknown.

Nuclear activation of proteins, like p38 and JNK, mediating stress response, is transient as their sustained activation may cause apoptosis [37, 38]. One mechanism of signal termination is receptor internalization and degradation. After binding to IL-1, although the receptor is internalized [39], it is not clear whether signaling terminates as it is found that IL-1 bound with the receptor accumulates inside nucleus after internalization without degradation [40] and evidence from signaling of other molecules suggests that signaling continues by receptor-ligand complex in endocytosed vesicles [41]. Thus, there is a possibility of IL-1 signaling to be sustained however it is known that p38 activation by IL-1 is transient and reaches a basal level in an hour in sustained presence of the cytokine [9]. Yet, the mechanism underlying basal activity remains unknown.

**Fig 1:**
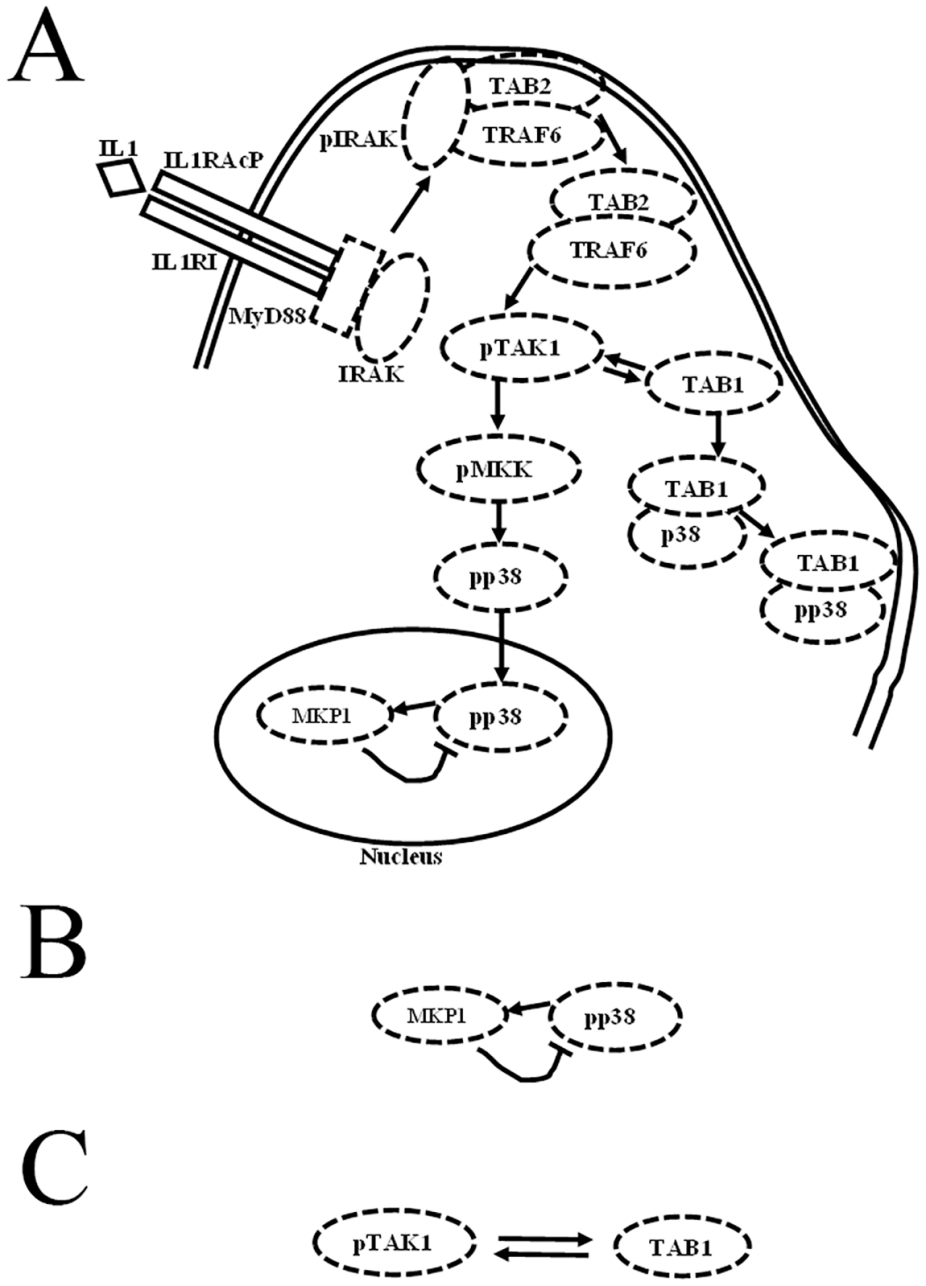
Interleukin-1 induced p38α activation network. (A) The protein-protein interactions in IL-1/p38 network have been shown. (B) The negative feedback loop between MKP1 and pp38. (C) The positive feedback loop between pTAK1 and TAB1.

While p38 is activated by MKK3/6 in a TAB2 dependent manner, it is dephosphorylated by a MAP kinase specific phosphatase, MKP1, and the active p38 increases MKP1 at a post-transcriptional level [42], creating a negative feedback loop (Fig 1A and 1B). Further, it has been shown that p38 can be activated by TAB1, independent of TAB2 and MKK3/6, although TAB1 activated p38 is sequestered in the cytoplasm [43].

In this study, we hypothesize that there is a positive feedback between TAB1 and pTAK1 (Fig 1A and 1C). While TAB1 phosphorylates TAK1 independent of TAB2, pTAK1 induces production of TAB1 at a post-transcriptional level. The study predicts that IL-1 induces a nuclear pulse of pp38 whose amplitude is primarily controlled by MKP1 through the negative feedback loop. Further, it predicts that IL-1 induces a cytoplasmic/membrane p38 response, which is primarily controlled by TAB1. Moreover, under sustained stimulation and in absence of receptor degradation, concentrations of active nuclear and membrane p38 return to their basal levels, which are controlled by TAB1 and MKP1 through combination of the positive and the negative feedback loops. In addition, the study found that the nuclear pulse is insensitive to a range of IL-1 concentration, suggesting robustness of the signaling network. Thus, the analysis addresses two fundamental questions about p38 activation by IL-1, namely, the mechanism underlying transient nuclear response and that responsible for the basal p38 activity.

## Results

### Interleukin-1 causes localization of active p38α to the nucleus and the membrane

**Fig 2:**
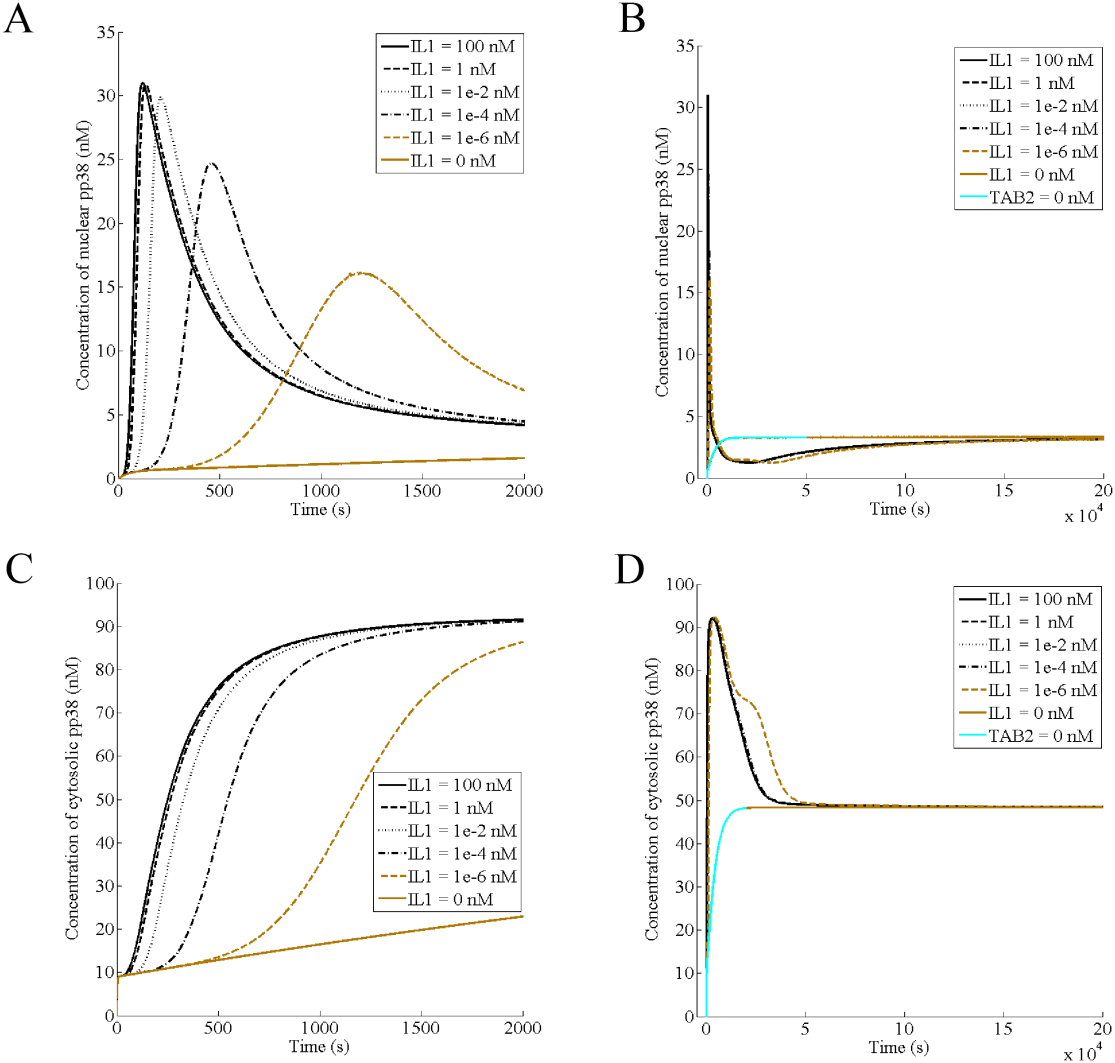
Interleukin-1 causes localization of active p38α to the nucleus and the membrane. IL-1 concentration has been varied as indicated. (A) The acute phase of the nuclear phospho-p38 in presence of IL-1 (B) Attainment of the steady state by the nuclear pp38 in presence of IL-1 (C) The transient phase of the cytosolic/ membrane phospho-p38 in presence of IL-1 (D) Attainment of the steady state by the cytosolic/membrane pp38 in presence of IL-1.

We constructed a model of p38 activation by IL1 using the Michaelis—Menten kinetics for all enzymatic reactions and the law of mass action for all protein-protein interactions. The rate constants for all the reactions and initial concentrations of the proteins are assumed. The model predicts the behavior of p38α under various conditions such as different concentrations of IL1 and cellular levels of various proteins. Toward this goal, first, we studied the effect of stimulation by IL-1on p38 activation. Addition of 100 nM of IL-1 rapidly translocates active pp38 to the nucleus, reaching a peak at t = 120s (Fig 2A). Then, its concentration decreases, reaching the basal level (IL1 = 0 in Fig 2B) at around t = 3724s. Thereafter, it stays around the basal level and finally reaches a steady state while IL-1 is continuously present (Fig 2B). The steady state achieved in presence of IL-1 is the same as the basal activity. Thus, the study shows two phases of p38 nuclear response: (i) an acute phase (ii) a near-constant phase. Reducing IL-1 concentration delays the first phase and reduces its peak (Fig 2A). However, for the concentrations of 100 nM and 1nM, the responses are almost identical; suggesting that in this range, varying the cytokine concentration may not affect the dynamics. IL-1 also causes pp38 to localize to membrane/cytoplasm. While the nuclear response is for a short duration, the cytosolic response is like a step change (Fig 2C). As in case of the nuclear fraction, decreasing IL-1 concentration delays the activation of the cytosolic fraction however it does not decrease its amplitude, unlike the nuclear response. After the transient phase, concentration of the cytosolic pp38 declines, reaching the basal level (Fig 2D). Similar to the nuclear activation, the membrane/cytosolic pp38 dynamics is almost identical for IL1 concentrations in the range of 100 nM-1 nM.

### Interleukin-1 causes rapid activation of MAP kinase kinase

**Fig 3:**
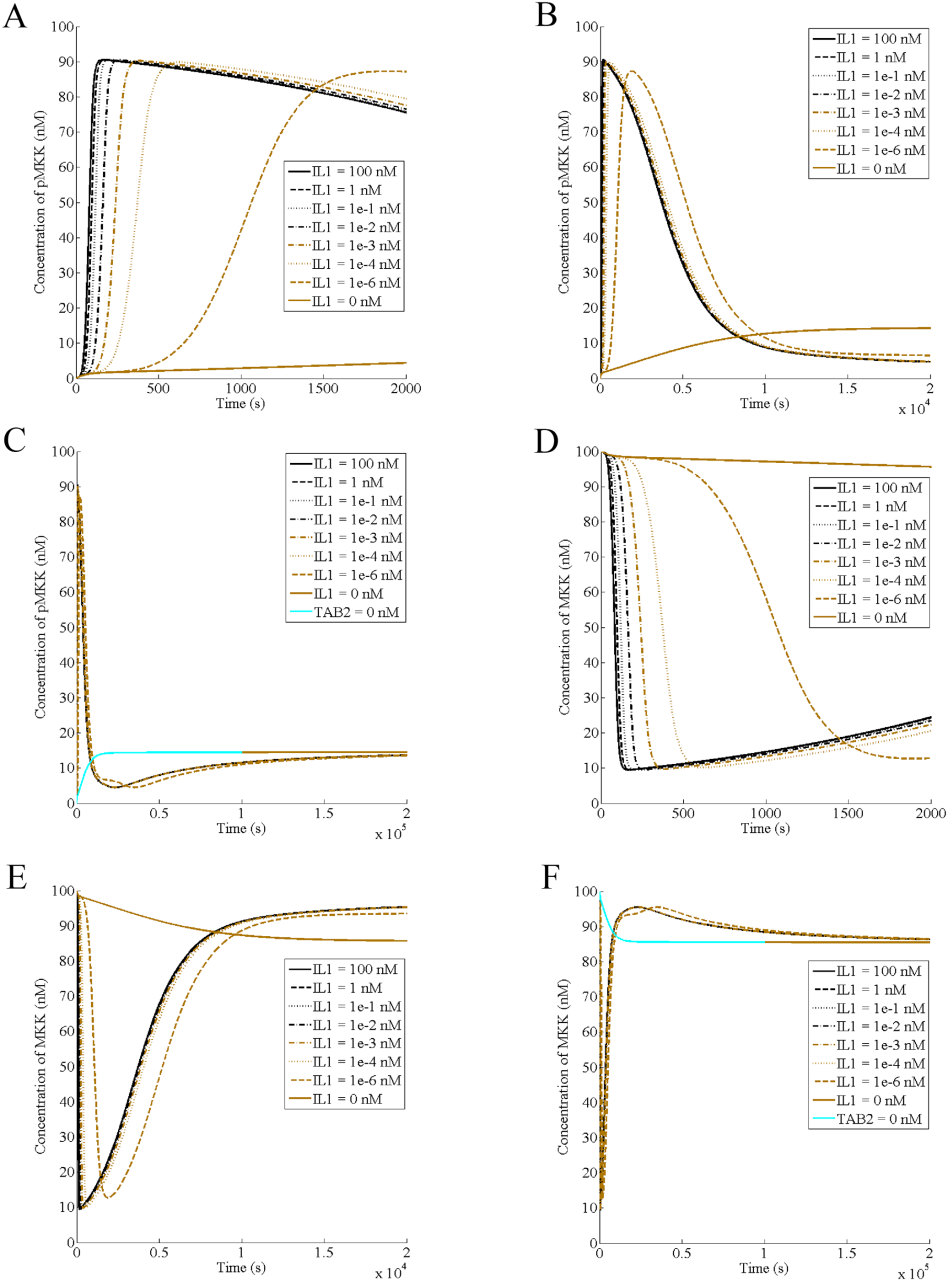
Interleukin-1 causes rapid activation of MAP kinase kinase. IL-1 concentration has been varied as indicated. (A) The activation phase of pMKK (B) The deactivation phase of pMKK (C) Attainment of the steady state by pMKK in presence of IL-1 (D) Dynamics of the inactive MKK during the activation phase of pMKK (E) Dynamics of the inactive MKK during the deactivation phase of pMKK (F) Attainment of the steady state by the inactive MKK in presence of IL-1.

Next, we predict the dynamics of MAP kinase kinase under IL-1 stimulation. Active MKK is present at a basal level (Fig 3C) and addition of IL-1 causes its further activation. Initially, concentration of pMKK increases at an increasing rate, reaching a point of inflection. Then, it slows to reach a maximum value (Fig 3A). After the maxima, pMKK starts to decrease, following a sigmoid curve (Fig 3B). Similar to the dynamics of nuclear pp38, after the deactivation phase, active MKK stays near the basal level and ultimately reaches a steady state, which is same as the basal level (Fig 3C). Like the nuclear and the membrane pp38, pMKK tends to reach a basal activity for all IL1 concentrations while the cytokine is present. The dynamics of unphosphorylated MKK is similar but opposite of that of pMKK since the total amount of the enzyme is a constant (Fig 3D, 3E and 3F). Thus, dynamics of pMKK consists of an activation phase, a deactivation phase, and a near-basal activity phase, which is followed by the steady state.

### TAB1 affects the steady states of the nuclear pp38, cytosolic pp38, and pMKK

**Fig 4:**
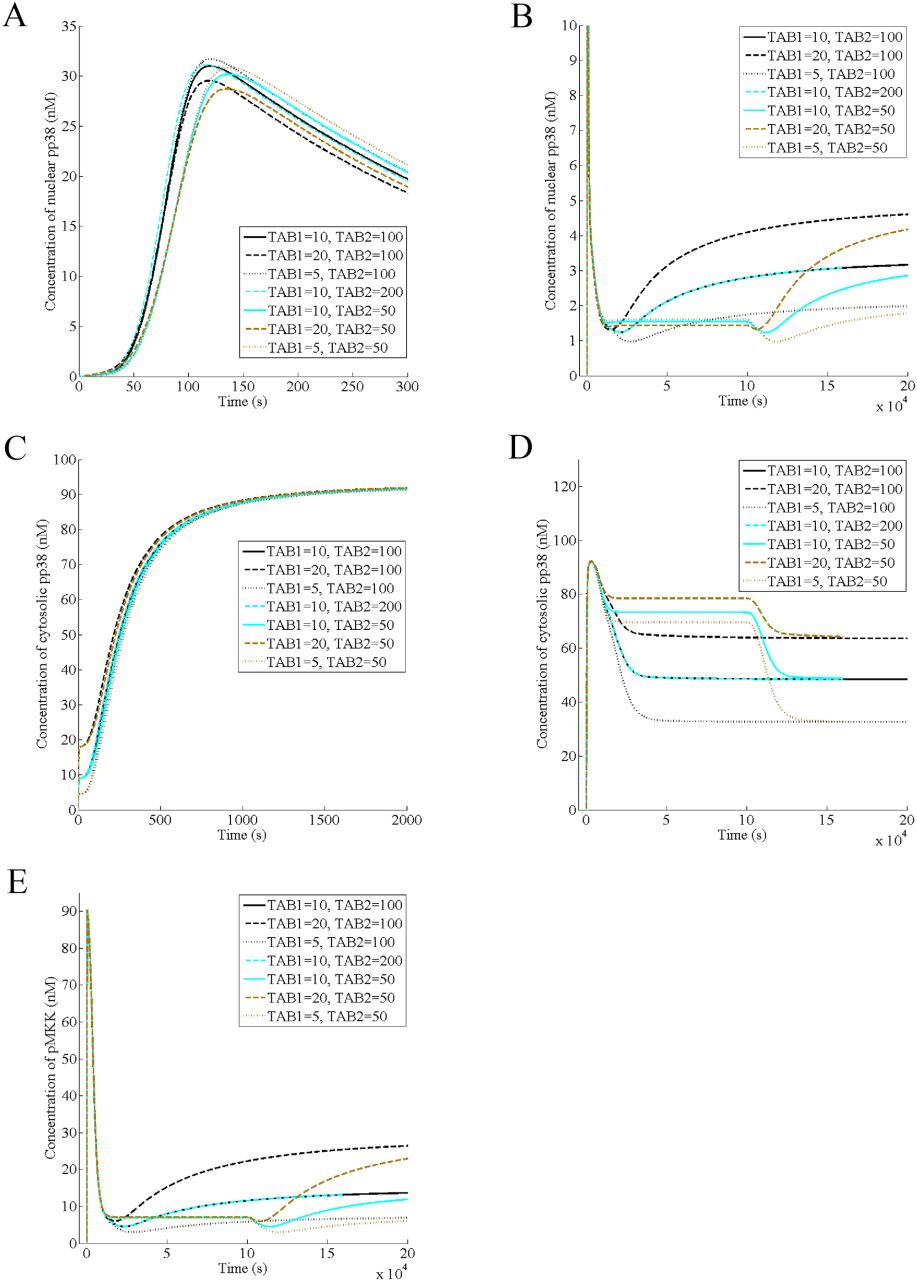
TAB1 affects the steady states of the nuclear pp38, cytosolic pp38, and pMKK. It is assumed that the normal levels of TAB1 and TAB2 in a wild type cell are 10 nM and 100 nM respectively (A) Effect of variation of TAB1 and TAB2 on the acute phase of the nuclear pp38 in presence of 100 nM of IL-1 (B) Effect of variation of TAB1 and TAB2 on the steady state of the nuclear pp38 in presence of 100 nM of IL-1 (C) Effect of variation of TAB1 and TAB2 on the transient phase of the cytosolic/membrane pp38 in presence of 100 nM of IL-1 (D) Effect of variation of TAB1 and TAB2 on the steady state of the cytosolic/membrane pp38 in presence of 100 nM of IL-1 (E) Effect of variation of TAB1 and TAB2 on the dynamics of pMKK in presence of 100 nM of IL-1.

Next, we predict the effect of varying the cellular level of TAB1 on the dynamics of nuclear pp38, cytosolic pp38 and pMKK. Toward this end, we found that decreasing TAB1 increases the amplitude of the acute phase of the nuclear pp38 by a small amount implying that the acute phase is not controlled primarily by TAB1 (Fig 4A). In contrast, increasing TAB1 increases the steady state of nuclear pp38, suggesting that its steady state is governed by TAB1 (Fig 4B). Like TAB1, TAB2 has a minor effect on the amplitude, which delays with decrease in the protein level (Fig 4A), although it has no effect on the nuclear pp38 steady state (Fig 4B). Like its effect on the steady state of the nuclear response, increasing TAB1 increases only the steady state of the cytoplasmic/membrane pp38 while TAB2 has no effect on either the transient phase of the cytoplasmic pp38 or its steady state (Fig 4C and 4D). Regarding pMKK activation, neither TAB1 nor TAB2 has any effect on either the activation or the deactivation phase of pMKK while its steady state increases with increase in TAB1 (Fig 4E). Thus, TAB1 affects the steady states of nuclear pp38, cytosolic pp38, and pMKK. Increasing TAB1 has a small yet negative effect on the amplitude of the nuclear pp38 since it takes a larger fraction of p38 and converts it to the cytosolic pp38. In contrast, increasing TAB1 has a strong positive effect on the cytosolic pp38 since TAB1 binding is required to generate membrane/cytoplasmic response. Further, it can be inferred that TAB1 affects the steady states of nuclear pp38, cytosolic pp38 and pMKK through the positive feedback loop between pTAK1 and TAB1.

### MKP1 negatively regulates the acute phase of the nuclear pp38 as well as its steady state

**Fig 5:**
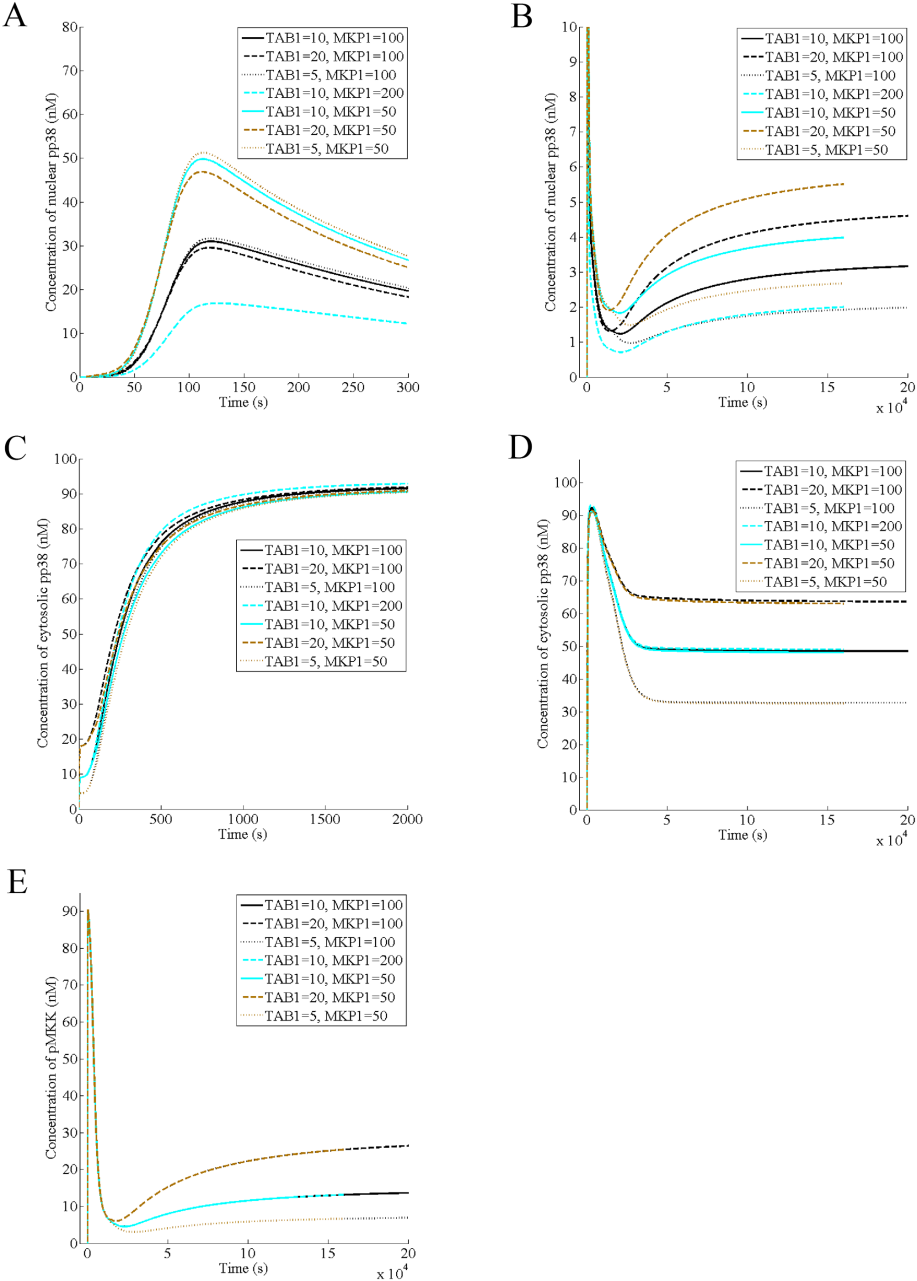
MKP1 negatively regulates the acute phase of the nuclear pp38 as well as its steady state. It is assumed that the normal levels of TAB1 and MKP1 in a wild type cell are 10 nM and 100 nM respectively. (A) Effect of variation of TAB1 and MKP1 on the acute phase of the nuclear pp38 in presence of 100 nM of IL-1 (B) Effect of variation of TAB1 and MKP1 on the steady state of the nuclear pp38 in presence of 100 nM of IL-1 (C) Effect of variation of TAB1 and MKP1 on the transient phase of the cytosolic/membrane pp38 in presence of 100 nM of IL-1 (D) Effect of variation of TAB1 and MKP1 on the steady state of the cytosolic/membrane pp38 in presence of 100 nM of IL-1 (E) Effect of variation of TAB1 and MKP1 on the dynamics of pMKK in presence of 100 nM of IL-1.

Next, we predict the effect of varying the cellular level of MKP1 on the dynamics of nuclear pp38, cytosolic pp38 and pMKK. Toward this end, we found that decreasing MKP1 level increases the amplitude of nuclear pp38 independent of TAB1 while for a fixed amount of MKP1, decreasing TAB1 increases the amplitude (Fig 5A), suggesting that MKP1 primarily regulates the acute phase of nuclear pp38. Like its effect on the amplitude, decreasing MKP1 increases the steady state of the nuclear pp38. In contrast, increasing TAB1 increases the steady state, suggesting that both TAB1 and MKP1 play important roles in modulating the steady state of the nuclear response (Fig 5B). In case of the cytosolic pp38, MKP1 affects its transient phase in a minor way, increasing the amplitude with increase in the protein (Fig 5C). Unlike TAB1, which affects the steady state of the cytosolic pp38 strongly, MKP1 has no significant effect on it (Fig 5D), implying a minor role of MKP1 in modulating the cytosolic response. Further, neither TAB1 nor MKP1 has any effect on either the activation or the deactivation phases of pMKK while its steady state depends on the TAB1 but not on MKP1 (Fig 5E), which is expected since MKP1 is downstream of pMKK. Thus, MKP1 governs the acute phase of nuclear pp38 while its steady state is controlled by both TAB1 and MKP1. Further, MKP1 has no significant effect on the dynamics of either the cytosolic pp38 or pMKK. The importance of MKP1 in the acute phase of the nuclear pp38 suggests a role of the negative feedback between MKP1 and nuclear pp38. Since the nuclear pp38 increases MKP1 over its unstimulated level, MKP1 ultimately restricts nuclear pp38 increase, reaching a peak. Since IRAK is degraded in presence of IL1 and not produced, causing termination of signaling, peak is not a stable point. Thus, nuclear pp38 concentration decreases along with MKP1, generating the acute phase. Interestingly, nuclear pp38 does not decrease to zero but to a steady state, which is the same as the basal level (Fig 2B).

### Effect of variation of TAB2 on the nuclear pp38, cytosolic/membrane pp38 and pMKK dynamics

**Fig 6:**
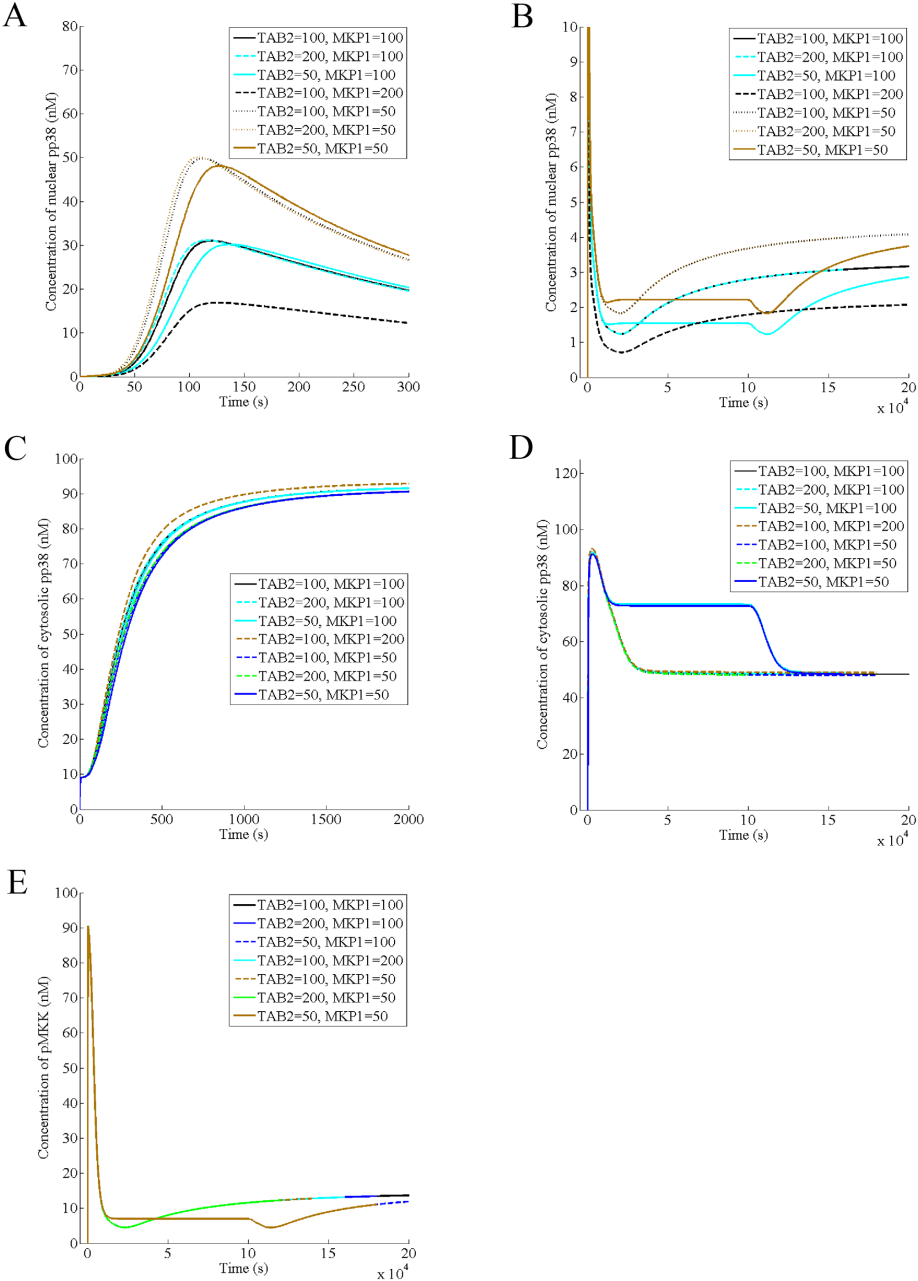
Effect of variation of TAB2 on the nuclear pp38, cytosolic/membrane pp38 and pMKK dynamics. It is assumed that the normal level of TAB2 and MKP1 in a wild type cell is 100 nM (A) Effect of variation of TAB2 and MKP1 on the acute phase of the nuclear pp38 in presence of 100 nM of IL-1(B) Effect of variation of TAB2 and MKP1 on the steady state of the nuclear pp38 in presence of 100 nM of IL-1 (C) Effect of variation of TAB2 and MKP1 on the transient phase of the cytosolic pp38 in presence of 100 nM of IL-1 (D) Effect of variation of TAB2 and MKP1 on the steady state of the cytosolic pp38 in presence of 100 nM of IL-1 (E) Effect of variation of TAB2 and MKP1 on the dynamics of pMKK in presence of 100 nM of IL-1.

Then, we predict the effect of varying the cellular level of TAB2 on the dynamics of nuclear pp38, cytosolic pp38 and pMKK and found that decreasing MKP1 increases the amplitude of the acute phase of nuclear pp38 regardless of TAB2 amount however decreasing TAB2 delays the amplitude only for a fixed level of MKP1, implicating MKP1 rather than TAB2 as the prime modulator of the nuclear p38 response (Fig 6A). Although MKP1 affects the steady state of the nuclear response, TAB2 has no effect on it unlike TAB1 (Fig 6B). Similarly, TAB2 has no effect on either the transient phase or the steady state of the cytosolic pp38 (Fig 6C and 6D). Further, TAB2 has no effect on both the activation and the deactivation phases of pMKK or its steady state (Fig 6E). Thus, lower cellular level of TAB2 delays the acute phase of the nuclear pp38, similar to the effect of lowering the concentration of IL1 (Fig 2A), suggesting a role of TAB2 in transduction of the extracellular signal.

### Roles of TAB1, TAB2, and MKP1 in the basal activities of the nuclear pp38, cytosolic pp38 and pMKK

**Fig 7:**
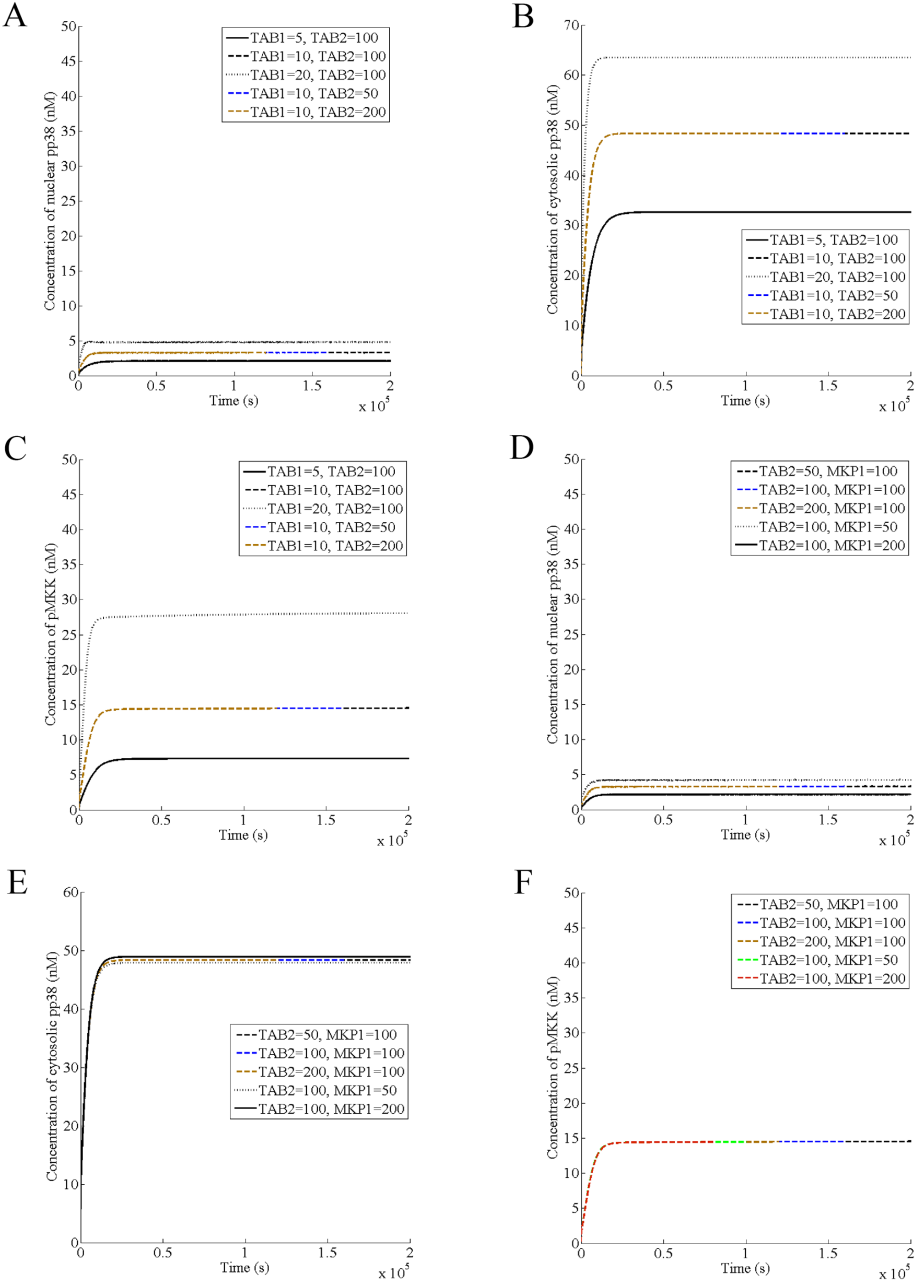
Roles of TAB1, TAB2, and MKP1 in the basal activities of the nuclear pp38, cytosolic pp38 and pMKK. It is assumed that the normal levels of TAB1, TAB2, and MKP1 are 10 nM, 100 nM, and 100 nM respectively. The amount of these proteins has been varied by varying their production rates in absence of IL-1. (A) and (D) basal activity of the nuclear pp38, (B) and (E) basal activity of the cytoplasmic pp38, (C) and (F) basal activity of pMKK.

Next, we predict the effect of varying the cellular level of TAB1, TAB2, and MKP1 on the dynamics of the nuclear pp38, cytosolic pp38, and pMKK in absence of IL-1 and found that increasing TAB1 increases the basal activity of the nuclear pp38, cytosolic pp38, and pMKK while TAB2 has no effect on it (Fig 7A, 7B and 7C). Further, decreasing MKP1 increases the basal level of the nuclear pp38 and decreases the basal activity of the cytosolic pp38 in a minor way while the basal activity of MKK is unaffected by MKP1 (Fig 7D, 7E and 7F), suggesting that TAB1 and MKP1 together govern the basal activity of the nuclear pp38 and TAB1 alone controls the basal levels of the cytosolic pp38 and pMKK. The hypothesized positive feedback loop between TAB1 and pTAK1 causes them to mutually regulate the cellular level of TAB1 and the activity of pTAK1, interlocking their levels and generating the basal activity of MKK. Thus, TAB1 through the positive feedback loop and MKP1 through the negative feedback loop together generate the basal level of the nuclear pp38. On the other hand, basal level of the cytosolic pp38 is controlled mainly by TAB1 through its direct binding with p38 and p38 autophosphorylation. Further, MKP1 affects the basal level of the cytosolic pp38 in a minor way by converting nuclear pp38 to p38.

### TAB1 is critical for the cytosolic pp38 response while TAB2 is required for the transmission of IL-1 signal and MKP1 governs the nuclear response along with TAB1

**Fig 8:**
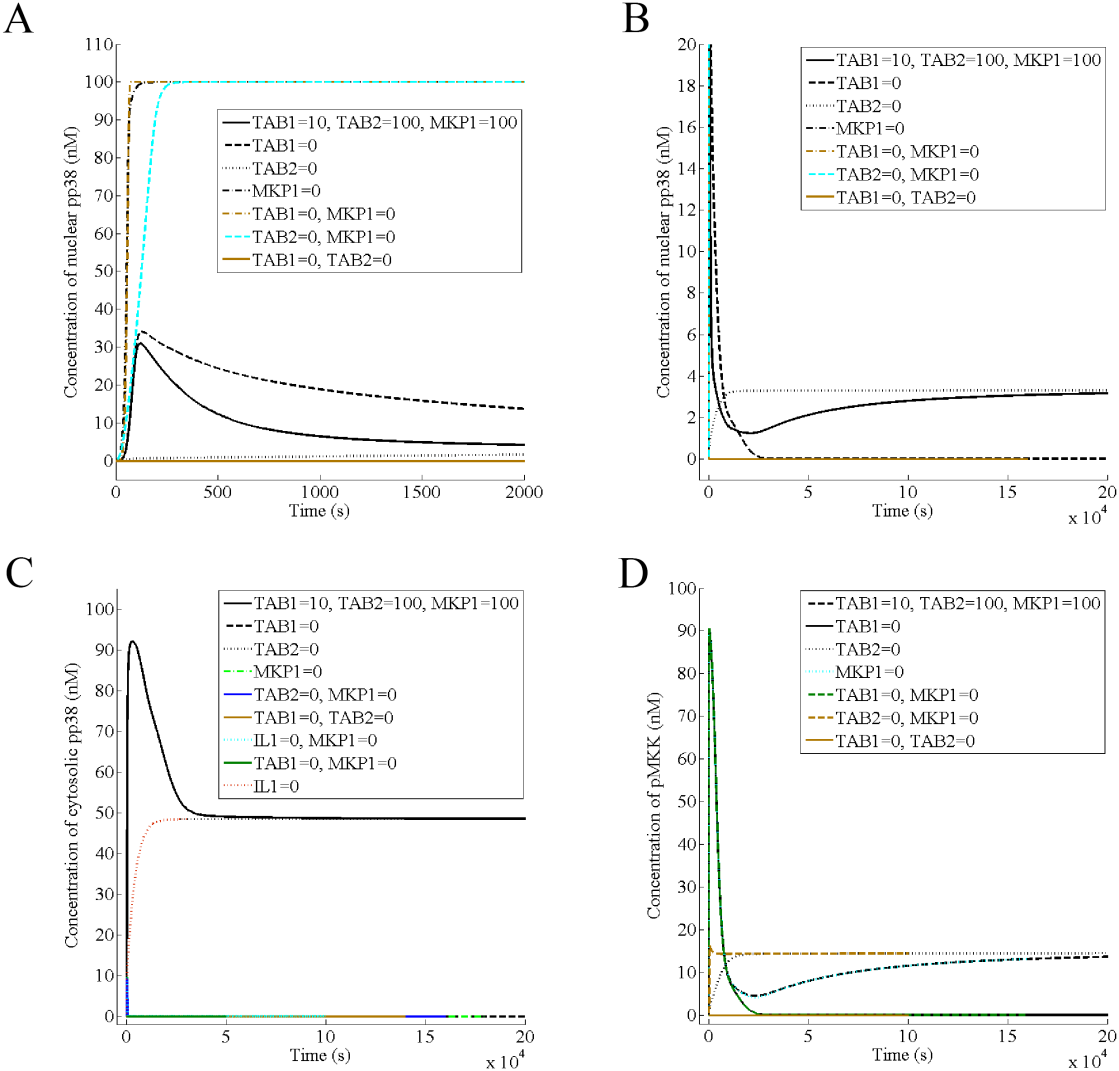
TAB1 is critical for the cytosolic pp38 response while TAB2 is required for the transmission of IL-1 signal and MKP1 governs the nuclear response along with TAB1. It is assumed that the normal levels of TAB1, TAB2, and MKP1 are 10 nM, 100 nM, and 100 nM respectively. The amount of these proteins has been abolished by setting their production rates to zero. (A) The acute phase of the nuclear phospho-p38 in presence of 100 nM of IL-1 (B) Attainment of the steady state by the nuclear pp38 in the presence of 100 nM of IL-1 (C) Response of the cytoplasmic phospho-p38 in presence of 100 nM of IL-1 (D) Dynamics of pMKK in presence of 100 nM of IL-1.

Next, we looked for the proteins, which are critical for IL-1 signal transduction and pp38 responses by deletion experiments in the mathematical model. First, we looked at the effect of TAB1 and found that removing TAB1 broadens the acute phase of the nuclear pp38 (Fig 8A) and reduces its steady state to zero (Fig 8B). Thus, in absence of TAB1, the transient phase of the nuclear response remains while its steady state becomes zero, suggesting that TAB1 controls the steady state (Fig 8A). Moreover, removing both TAB1 and TAB2 nullifies the activation of nuclear pp38 completely, suggesting that the absence of both proteins will render cells nuclear pp38 null (Fig 8A and 8B). In continuation, we looked at the effect of TAB1 on the cytosolic pp38 and found that removing TAB1 nullifies the cytosolic response completely (Fig 8C), suggesting that TAB1 is critical for the membrane/cytosolic p38 activation.

Next, we predict the effect of removing MKP1 from the cell and found that absence of MKP1 causes constitutive activation of the nuclear pp38, suggesting that MKP1 controls the acute phase of the nuclear response (Fig 8A and 8B). In absence of both TAB1 and MKP1, all of p38 is phosphorylated since the phosphatase is absent and none is sequestered in cytoplasm since TAB1 is absent, causing constitutive activation of nuclear pp38 (Fig 8A).

In the above, we looked for the proteins, which regulate the acute phase and the steady state of pp38 responses. Next, we investigated the protein, which is critical for IL1 signal transduction by looking at MKK and p38 activation. If deletion of a protein causes the dynamics of MKK and p38 in the presence of IL1 to be the same as that in the absence of IL1, the protein can be inferred to have a role in IL1 signal transduction. Toward this end, we found that MKP1 has no effect on pMKK dynamics (Fig 8D) and removal of TAB1 nullifies only the steady state of pMKK (Fig 8D). On the other hand, removing TAB2 abolishes the IL1 response (Figs 2B, 2D, 3C and 8C), suggesting that while TAB1 maintains the steady state of pMKK, TAB2 is required for the transmission of IL1 signal.

## Discussion

We constructed a mathematical model of p38 activation by IL-1, incorporating known protein-protein interactions in the IL-1/p38 signaling network. The model includes an already established negative feedback loop between nuclear pp38 and MKP1 and an assumed positive feedback loop between pTAK1 and TAB1. Further, it incorporates both the nuclear and the cytosolic activation of p38. The model predicts that in absence of receptor degradation and sustained presence of IL1, concentrations of both the nuclear and the cytosolic pp38 return to their basal levels, which depend on protein levels of TAB1 and MKP1 in the cell. The nuclear response consists of an acute phase of about an hour, which was found to be unaltered when cytokine concentration was decreased in a range of 100-fold. Since the upper limit of the cytokine concentration range was assumed to be the same as the IL1 receptor concentration, the unaltered response at the lower limit of IL1 concentration suggests that the IL1/p38 signaling network is biochemically robust. It is possible that the dissociation rate constant of a specific protein-protein interaction is small and the downstream reactions are fast so that a small number of active upstream complexes may activate a large number of downstream effectors. The exact basis of the predicted robustness remains to be investigated in future.

Toward the experimental verification of the predicted time course of nuclear pp38, Taichiro et al. [44] studied the dynamics of p38 activity in both individual and population of cells in response to IL-1 and found that in the individual cells, the dynamics consists of an initial acute phase, followed by several oscillatory peaks, which continued for upto 8 h under sustained IL-1 stimulation. On the other hand, in the population of cells, the dynamics consist of an initial transient phase of 1 h duration followed by a near basal activity, consistent with our model. They explained the near basal activity based on the asynchronocity of oscillations in the individual cells. It is already established that gene expression in individual cells is stochastic in nature [45]. Stochasticity of DNA-transcription factor interaction, coupled with different resistances of kinetic rate and cytoplasmic-nuclear transport can also cause oscillations. We will incorporate these aspects in signal transduction models in future.

Our model predicted the effect of MKP1, TAB1 and TAB2 on the acute phases of the nuclear and the cytosolic pp38 as well as their basal activities. Regarding MKP1, it predicts that MKP1 decreases the peak of the transient phase of the nuclear pp38. Experimentally, it has been established that MKP1 is an inhibitor of p38 activity [46, 47]. Besides decreasing the peak, MKP1 also decreases the basal activity of nuclear pp38. In agreement with our prediction, Rastogi et al. [48] found that BAL cells from patients with sarcoidosis, a systemic inflammatory disorder, in which MKP1 expression is attenuated, exhibited higher basal p38 activity.

Regarding TAB1, De Nicola et al. [49] and Ge et al. [50] showed that its binding to p38α causes autophosphorylation of p38α. Further, in cardiac myocytes and perfused mouse hearts, TAB1 activates p38 independent of MAPKK. Moreover, De Nicola et al. found that abolishing p38-TAB1 interaction abolishes cardiac toxicity. This interaction may be a target of drug therapy which could circumvent drawbacks of blocking p38 kinase activity by pharmacological inhibitors [49]. Besides autophosphorylation, p38α can be activated by TAB1, independent of TAB2-TRAF6, through direct phosphorylation and activation of TAK1 by the TAB1-TAK1 interaction [51, 52]. Thus, there are two ways p38 can be activated by TAB1: i. through p38α-TAB1 interaction and autophosphorylation of p38α in a MKK3/6 independent manner ii. through direct interaction of TAB1-TAK1 followed by autophosphorylation and activation of TAK1 resulting in MKK3/6 activation. We have assumed that in MKK dependent activation, pp38 is shuttled to the nucleus, which is consistent with Gong et al.’s finding that nuclear translocation of p38 is phosphorylation dependent [53]. We predict that varying TAB1 has no significant effect on the amplitude of the nuclear pp38 (Fig 4A) and in absence of TAB1, the acute phase of the nuclear pp38 remains (Fig 8A), which is in agreement with Inagaki et al. [54] who show that p38 is activated by IL-1 in MEFs with or without TAB1. Regarding the effect of TAB1 on the cytosolic pp38, we predict that TAB1 is critical for membrane/cytoplasmic response, which is consistent with Lu et al’s finding that TAB1 segregates p38 in the cytosol and prevents the expression of inflammatory and cardiac marker genes or changes in cellular morphology [43].

Regarding TAB2, we predict that it does not affect the steady state/basal activity of either the nuclear or the cytosolic pp38. It is known that TAK1 is essential for IL1 signaling [55]. Further, Takaesu et al. have shown that TAB2 does not have any enzymatic activity towards TAK1 [6]. Thus it can be inferred that TAB2 expression level will not affect the basal activity of p38, consistent with our prediction. Takaesu et al. have also shown that TAB2 links TRAF6 to TAK1, an interaction necessary for IL1 induced TAK1 activation [6, 56]. Thus, deletion of TAB2 will abolish IL1/p38 signaling, resulting in only the basal p38 activity, consistent with our prediction. In the model, we have not considered the role of TAB3, which is a homolog of TAB2 [57]. Thus, there may be redundancies in TAB2-TAK1 interaction.

In the model, we hypothesized that there is a positive feedback loop between pTAK1 and TAB1 (Fig 1C). Toward the proof of the hypothesis, it has been shown that TAB1 direct binding causes TAK1 activation through oligomerization and autophosphorylation [51, 52], establishing the backward arm of the loop. The forward arm, pTAK1 increases TAB1 at a post-transcriptional level, is not well established. In support of our hypothesis, Omori et al. show that in epidermis of TAK1-null mice, TAB1 expression has attenuated, suggesting that the TAK1-TAB1 interaction stabilizes TAB1 [51]. Further, Pathak et al. show that TAB1 is O-GlcNAcylated at Ser395 position and IL1 increases the amount of O-GlcNAcylated TAB1 [58]. In addition, Pathak et al show that O-GlcNAcylation of TAB1 is required for full phosphorylation and activation of TAK1. From both these studies, it can be inferred that O-GlcNAcylation of TAB1, which is induced in a pTAK1 dependent manner, may increase stability of TAB1.

Besides stress and inflammation, in which nuclear activity of p38 is important, MKK independent p38 activation and the basal p38 activity have importance in ischemic injury and diabetes. Increased p38 basal activity reduces contractility of cardiac myocytes through desensitization of contractile myofilaments’ response to Ca^++^ [59]. In contrast, pharmacological experiments show that p38 activation in mouse heart through short term hibernation or low-flow ischemia did not correlate with contractile deficit [60]. Taken together, these studies point toward a sustained role of p38 rather than the transient p38 activation in causing the contractile deficit. Further, ischemia injury has been shown to activate p38. However, deletion of MKK3 did not have considerable effect on p38 activation in ischemia, leading to the conclusion that TAB1 mediated autophosphorylation, instead of MKK3, may be responsible for p38 activation in ischemic heart [61]. Since, the experiment did not directly vary TAB1 amount in ischemic heart, it can be inferred that induction of ischemia may have induced TAB1, leading to the increased p38 activation. It will be interesting to see whether TAK1 phosphorylation and TAB1 level increases in ischemic heart. Interestingly, hyperglycemic condition increased O-GlcNAcylation of TAB1 [58]. Since enhanced basal activity of p38 has been correlated with type 2 diabetes [62], it may be possible that increased O-GlcNAcylation of TAB1 is the cause of both reduced contractility of cardiac myocytes and hyperglycemia.

In conclusion, the model predicts that IL1 causes a robust p38α activation in the nucleus and in the cytoplasm. Using a set of assumptions, we predict the effect of variation of TAB1, TAB2, and MKP1 on the nuclear and the cytoplasmic pp38 transient phases and their steady states. We predict that MKP1 controls the amplitude of the acute phase of the nuclear pp38, suggesting a role of MKP1-pp38 negative feedback loop. Further, we predict that TAB1 is essential for the cytoplasmic pp38 response. In addition, we predict that both TAB1 and MKP1 regulate the steady states/basal activities of the nuclear and the cytoplasmic pp38, suggesting a role of pTAK1-TAB1 positive feedback loop. Our study has implications for ischemia and diabetes.

## Materials and Methods

The signaling network orchestrating the nuclear and the membrane p38 activity in response to IL1 has been constructed as shown in Fig 1A. All phosphorylation/dephosphorylation reactions have been assumed to follow Michaelis—Menten kinetics. All phosphatases except MKP1 have been assumed to be in excess. Besides phosphorylation/dephosphorylation and binding reactions, the study includes ubiquitination and degradation of TAB2, TRAF6, pTAK1, and TAB2, following IL-1stimulation. It has been assumed that TAB2, TRAF6, TAK1, and TAB2 are continuously produced with a rate α (nM/s) and degraded with a rate constant β(s^-1^) so that their steady state level, α/β, is maintained in the cell. Further, we have considered that IRAK is rapidly degraded by IL-1 signaling [63] but not produced during the course of the stimulation. The reactions and their rate constants have been given in the supporting information section (S1 Text). The pre-stimulation protein levels have been assumed to be present in a wild type cell and have been given in supporting information section (S1 Text). Half-life of MKP1 has been taken as 40 min [64]. To study the effects of these proteins on the nuclear and the membrane/cytosolic p38 responses as well as on their basal activities, pre-stimulation amounts of the proteins have been varied by varying their production rate, α. To abolish a protein from the cell, pre-stimulation level of that protein as well as its production rate has been set to zero. The resulting set of ODEs has been solved using ode solver ODE113 of MATLAB 2012a. Although the study observes basal levels of active nuclear and membrane pp38 as well as that of pMKK, initial levels of the active proteins have been taken as zero for all IL-1 stimulation studies.

## Supporting Information

S1 Text Reactions, rate of reactions, rate constants, and initial concentrations. Reactions, rate of reactions, rate constants, and initial concentrations of the proteins, involved in the IL1/p38 network have been listed in the supporting information.
(DOC)
