# Supplementary material for: Model Predicts That MKP1 and TAB1 Regulate p38α Nuclear Pulse and Its Basal Activity through Positive and Negative Feedback Loops in Response to IL-1
Source: PLoS One. 2016 Jun 17;11(6):e0157572. doi: 10.1371/journal.pone.0157572 (PMC4912083; doi:10.1371/journal.pone.0157572)
Supplement: S1 Text — Reactions, rate of reactions, rate constants, and initial concentrations of the proteins, involved in the IL1/p38 network have been listed in the supporting information. (DOC) [file pone.0157572.s001.doc]

**S1 Text. Reactions, rate of reactions, rate constants, and initial concentrations**

**Reactions in the IL-1 induced p38 activation network**

$$I\text{L1 }+\text{ IL1Rl }\Leftrightarrow\text{ R}_{\text{L}}\text{; (1)}$$

$$\text{R}_{\text{L}}+\text{ IL1RAcP }\Leftrightarrow\text{ R}_{\text{LC}}\text{; (2)}$$

$$\text{R}_{\text{LC}}+\text{ MyD88 }\Leftrightarrow\text{ R}_{\text{LCM}}\text{; (3)}$$

$$\text{ pI}RAK\xrightarrow{}\text{IRAK; (5)}$$

$$TAB2+\text{ pIRAK }\Leftrightarrow\text{ pIRAK\_TAB2; (6)}$$

$$pIRAK\_TAB2+\text{ TRAF6 }\Leftrightarrow\text{ pIRAK\_TAB2\_TRAF6; (7)}$$

$$\text{pIRAK\_TAB2\_TRAF6}\xrightarrow{}\text{mTAB2\_TRAF6 }+\text{ pIRAKu; (8)}$$

$$\text{mTAB2\_TRAF6 }\xrightarrow{}\text{TAB2\_TRAF6; (9)}$$

$$\text{TAB2\_TRAF6 }\xrightarrow{}\text{uTAB2 }+\text{ uTRAF6; (10)}$$

$$\text{pTAK1 }\xrightarrow{}\text{uTAK1; (12)}$$

$$\text{pTAK2 }\xrightarrow{}\text{TAK1; (15)}$$

$$\text{pMKK }\xrightarrow{}\text{MKK; (17)}$$

$$TAB1+\text{ p38 }\Leftrightarrow\text{ mpp38; (20)}$$

$$\text{NULL }\xrightarrow{}\text{TAB2; (22)}$$

$$\text{NULL }\xrightarrow{}\text{TRAF6; (23)}$$

$$\text{NULL }\xrightarrow{}\text{TAK1; (24)}$$

$$\text{uTAK1 }\xrightarrow{}\text{NULL; (25)}$$

$$\text{uTAB2 }\xrightarrow{}\text{NULL; (26)}$$

$$\text{uTRAF6}\xrightarrow{}\text{NULL; (27)}$$

$$\text{p}IRAKu\xrightarrow{}\text{NULL; (28)}$$

**Rate of reactions**

$$\text{ r}_{1}=\text{ k}_{1}\text{ IL1. IL1Rl - k}_{\text{-1}}\text{R}_{\text{L}}\text{ }$$

$$\text{ r}_{2}=\text{ k}_{2}\text{ R}_{\text{L}}\text{. IL1RAcP - k}_{\text{-2}}\text{.R}_{\text{LC}}\text{ }$$

$$\text{ r}_{3}=\text{ k}_{3}\text{ R}_{\text{LC}}\text{. MyD88 - k}_{\text{-3}}\text{R}_{\text{LCM}}\text{ }$$

$$\text{r}_{4}=\text{ }\frac{\text{v}_{4}.\begin{matrix} \end{matrix}IRAK.\begin{matrix} \end{matrix}\text{R}_{\text{LCM}}}{\text{k}_{4}+IRAK}\text{ }$$

$$\text{r}_{5}=\text{ }\frac{\text{v}_{5}.pIRAK}{\text{k}_{5}+pIRAK}\text{ }$$

$$\text{ r}_{6}=\text{ k}_{6}\text{ TAB2. pIRAK - k}_{\text{-6}}\text{pIRAK\_TAB2 }$$

$$\text{ r}_{7}=\text{ k}_{7}\text{ . pIRAK\_TAB2. TRAF6 - k}_{\text{-7}}\text{.pIRAK\_TAB2\_TRAF6}$$

$$\text{r}_{8}=\text{k}_{8}\text{. pIRAK\_TAB2\_TRAF6 }$$

$$\text{r}_{9}=\text{k}_{9}\text{. mTAB2\_TRAF6 }$$

$$\text{ r}_{10}=\text{k}_{10}\text{. TAB2\_TRAF6 }$$

$$\text{r}_{11}=\text{ }\frac{\text{v}_{11}.\begin{matrix} \end{matrix}TAK1.\begin{matrix} \end{matrix}\text{TAB2\_TRAF6}}{\text{k}_{11}+TAK1}$$

$$\text{r}_{12}=\text{ k}_{12}\text{.pTAK1 }$$

$$\text{r}_{13}=\text{ }\alpha\text{-}\beta\text{. TAB1}+\frac{\text{v}_{13}.pTAK1}{\text{k}_{13}+pTAK1}$$

$$\text{r}_{14}=\text{ }\frac{\text{v}_{14}.\begin{matrix} \end{matrix}TAK1.\begin{matrix} \end{matrix}\text{TAB1}}{\text{k}_{14}+TAK1}$$

$$\text{r}_{15}=\text{ }\frac{\text{v}_{15}.pTAK2}{\text{k}_{15}+pTAK2}$$

$$\text{r}_{16}=\text{ }\frac{\text{v}_{16}.(pTAK1+pTAK2).MKK}{\text{k}_{16}+MKK}$$

$$\text{r}_{17}=\text{ }\frac{\text{v}_{17}.pMKK}{\text{k}_{17}+pMKK}$$

$$\text{ r}_{18}=\text{ }\frac{\text{v}_{18}.\begin{matrix} \end{matrix}p38.\begin{matrix} \end{matrix}pMKK}{\text{k}_{18}+p38}\text{ }$$

$$\text{r}_{19}=\text{ }\frac{\text{v}_{19}.\begin{matrix} \end{matrix}pp38\_n.\begin{matrix} \end{matrix}MKP1}{\text{k}_{19}+pp38\_n}\text{ }$$

$$\text{r}_{20}=\text{ k}_{20}\text{. TAB1. p38 - k}_{\text{-20}}\text{. mpp38 }$$

$$\text{r}_{21}=\text{ }\alpha_{2}\text{-}\beta_{2}\text{. MKP1}+\frac{\text{v}_{21}.pp38\_n}{\text{k}_{21}+pp38\_n}$$

$$\text{r}_{22}=\text{ }\alpha_{5}\text{-}\beta_{5}\text{. TAB2}$$

$$\text{r}_{23}=\text{ }\alpha_{4}\text{-}\beta_{4}\text{. TRAF6}$$

$$\text{r}_{24}=\text{ }\alpha_{3}\text{-}\beta_{3}\text{. TAK1}$$

$$\text{r}_{25}=\text{k}_{25}\text{. uTAK1 }$$

$$\text{r}_{26}=\text{k}_{26}\text{. uTAB2 }$$

$$\text{r}_{27}=\text{k}_{27}\text{. uTRAF6 }$$

$$\text{r}_{28}=\text{k}_{28}\text{. pIRAKu }$$

**Rate constants**

k1=0.01 nM^-1^s^-1^; k_1=0.0012 s^-1^;

k2=0.0001 nM^-1^s^-1^; k_2=0.0008 s^-1^;

k3=0.005 nM^-1^s^-1^; k_3=0.05 s^-1^;

v4=5 nM^-1^s^-1^; k4=50 nM;

v5=15 s^-1^; k5=500 nM;

k6=0.005 nM^-1^s^-1^; k_6=0.05 s^-1^;

k7=0.005 nM^-1^s^-1^; k_7=0.05 s^-1^;

k8=0.05 s^-1^;

k9=0.005 s^-1^;

k10=0.0005 s^-1^;

v11=0.15 nM^-1^s^-1^; k11=50 nM;

k12=0.0005 s^-1^;

α=0.01 nM s^-1^; β=0.001 s^-1^;

v13=0.5 s^-1^; k13=50 nM;

v14=0.02 nM^-1^s^-1^; k14=50 nM;

v15=15 s^-1^; k15=500 nM;

v16=0.15 nM^-1^s^-1^; k16=50 nM;

v17=15 s^-1^; k17=500 nM;

v18=0.5 nM^-1^s^-1^; k18=20 nM;

v19=5 nM^-1^s^-1^; k19=500 nM;

k20=0.005 nM^-1^s^-1^; k_20=0.05 s^-1^;

v21=1.0 s^-1^; k21=200 nM;

α2=2.888e-2 nM s^-1^; β2=2.888e-4 s^-1^;

α3=0.001 nM s^-1^; β3=0.00001 s^-1^;

α4=0.001 nM s^-1^; β4=0.00001 s^-1^;

α5=0.001 nM s^-1^; β5=0.00001 s^-1^;

k25=0.000012 s^-1^;

k26=0.000012 s^-1^;

k27=0.000012 s^-1^;

k28=0.000012 s^-1^;

**Initial concentration (nM)**

IL1R1 = 100;

R_L_ = 0;

IL1RAcP = 100;

R_LC_=0;

MYD88= 100;

R_LCM_= 0;

IRAK= 100;

pIRAK= 0;

TAB2= 100;

pIRAK_TAB2= 0;

TRAF6= 100;

pIRAK_TAB_TRAF6=0;

mTAB2_TRAF6= 0;

pIRAKu= 0;

TAB2_TRAF6= 0;

uTAB2= 0;

uTRAF6= 0;

TAK1=100;

pTAK1= 0;

uTAK1= 0;

TAB1= 10;

pTAK2= 0;

MKK= 100;

pMKK=0;

p38= 100;

pp38=0;

MKP1= 100;

mpp38= 0;

pIRAKu= 0;

pp38_n=0;

where,

IL1R1: Interleukin-1 receptor; R_L_ : Receptor-ligand complex; IL1RAcP : receptor accessory protein; R_LC_ : receptor-ligand-accessory protein complex; R_LCM_ :receptor-ligand-accessory protein-MyD88 complex; pIRAK: phosphorylated IRAK; pIRAK_TAB2: phosphorylated IRAK-TAB2 complex; pIRAK_TAB_TRAF6: phosphorylated IRAK-TAB2-TRAF6 complex; mTAB2_TRAF6: TAB2-TRAF6 complex at the membrane; IRAKu: ubiquitinated IRAK; TAB2_TRAF6: TAB2-TRAF6 complex in the cytoplasm; uTAB2: ubiquitinated TAB2; uTRAF6: ubiquitinated TRAF6; pTAK1: TAB2 dependent phosphorylated TAK1; uTAK1: ubiquitinated TAK1; pTAK2: TAB1 phosphorylated TAK1; MKK: MAP kinase kinase; pMKK : phosphorylated MAP kinase kinase; pp38 : phospho-p38; pp38_n : nuclear phospho-p38; mpp38 : cytoplasmic phospho-p38; pIRAKu : ubiquitinated phospho-IRAK;
